# Prospective approaches to gene therapy computational modeling – spotlight on viral gene therapy

**DOI:** 10.1007/s10928-023-09889-1

**Published:** 2023-10-17

**Authors:** Mary P Choules, Peter L. Bonate, Nakyo Heo, Jared Weddell

**Affiliations:** Early Development, New Technologies Group, Astellas, Northbrook, IL USA

## Abstract

Clinical studies have found there still exists a lack of gene therapy dose-toxicity and dose-efficacy data that causes gene therapy dose selection to remain elusive. Model informed drug development (MIDD) has become a standard tool implemented throughout the discovery, development, and approval of pharmaceutical therapies, and has the potential to inform dose-toxicity and dose-efficacy relationships to support gene therapy dose selection. Despite this potential, MIDD approaches for gene therapy remain immature and require standardization to be useful for gene therapy clinical programs. With the goal to advance MIDD approaches for gene therapy, in this review we first provide an overview of gene therapy types and how they differ from a bioanalytical, formulation, route of administration, and regulatory standpoint. With this biological and regulatory background, we propose how MIDD can be advanced for AAV-based gene therapies by utilizing physiological based pharmacokinetic modeling and quantitative systems pharmacology to holistically inform AAV and target protein dynamics following dosing. We discuss how this proposed model, allowing for in-depth exploration of AAV pharmacology, could be the key the field needs to treat these unmet disease populations.

## Introduction

It’s been almost 200 years since the start of the modern pharmaceutical industry. Since its inception, pharmaceutical companies have been primarily developing therapies to treat the symptoms of disease, not cure disease. Although some therapies, like insulin to treat diabetes, have been developed to treat the basis of disease, these therapies are short-acting and require chronic treatment over a patient’s lifetime. The pharmaceutical industry today is on the cusp of treating disease right at its heart – to curing some diseases completely. Gene therapy, which when you think about it, is the stuff of science fiction, targets the nucleus of a patient’s cells and uses the patient’s natural biochemistry to produce, turn on, or turn-off proteins that lead to disease. Imagine getting a single injection of a gene therapy product and then never needing another treatment for that disease *for the rest of your life*. This is the goal of gene therapy.

The “five rights” of health care are: the right patient, the right drug, the right time, the right dose, and the right route [1]. In many ways this adage is the pledge of pharmacokinetic modelers, regardless their area of specialty. However, with cell and gene therapies, the answer to many of these “five rights” can become muddled, especially at early phases of development. For this reason, systems pharmacology has become an essential tool to answer these critical questions. Although the area of application is still emerging with minimal regulatory guidance, it is important for the advancement of the community to offer guidance to those looking to utilize modeling to aid the development of gene therapies.

For this reason, the purpose of this review is to introduce gene therapy, to highlight the systems pharmacology models that have been developed (and not developed) for gene therapy, including physiological-based pharmacokinetic models, how these models have been used to support model-informed drug development, give recommendations on how to build these types of models, and to discuss the limitations and future related to systems pharmacology of gene therapy products.

### Overview of common gene therapy technologies

**Fig. 1:**
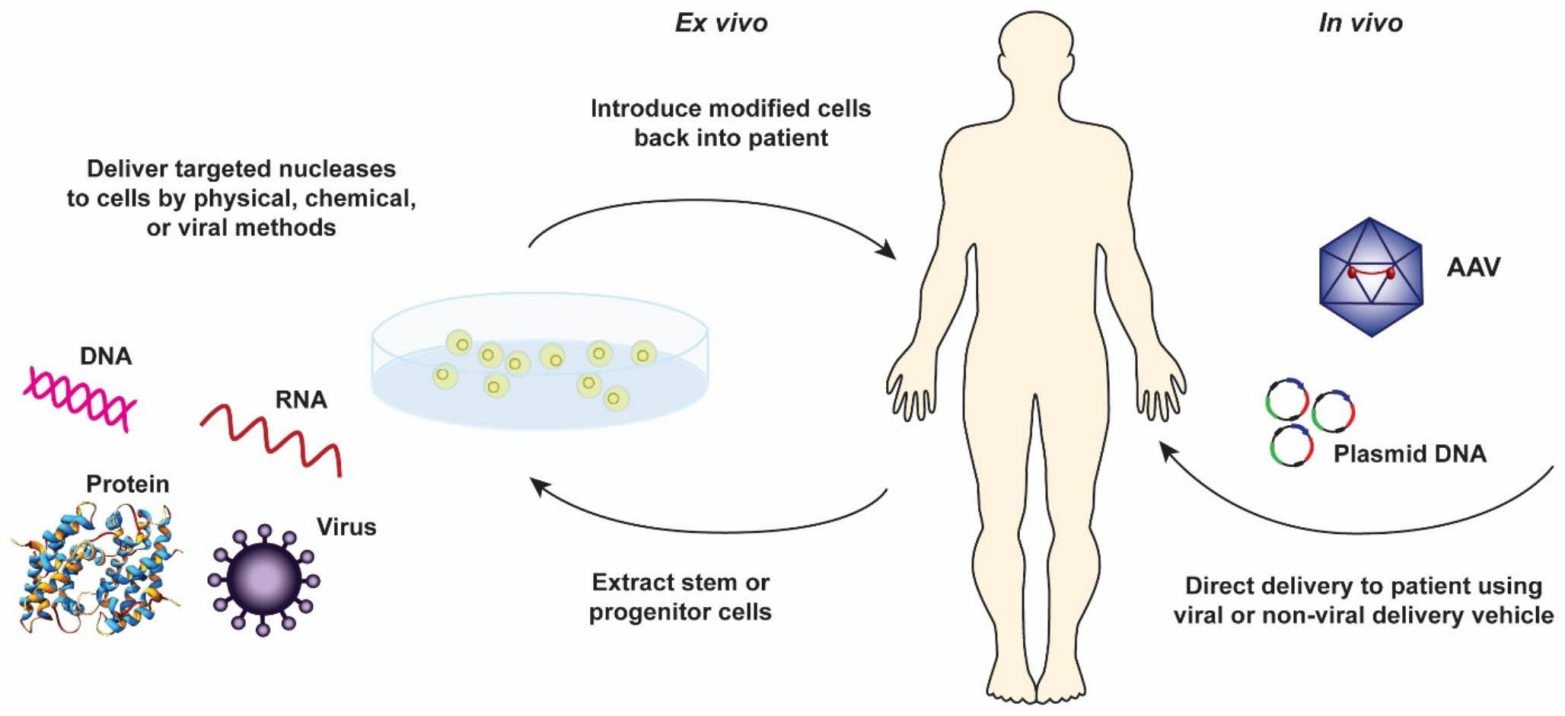
Visual representation of gene therapy types: ex vivo and in vivo therapies. Adapted from the FDA(https://www.fda.gov/biologicsbloodvaccines/cellulargenetherapyproducts/ucm573960.htm)

The definition of gene therapy according to the FDA is “the administration of genetic material to modify or manipulate the expression of gene product or to alter the biological properties of living cells for therapeutic use [2].” Gene therapies involve the administration of designated genetic material to the target tissues via a carrier or “vector.” The vector can take many forms but this review will focus on two types: viral-based and plasmid-based with more emphasis placed on adeno-associated virus (AAV) vectors. Vector delivery can also be broken down into in vivo and ex vivo as illustrated in Fig. 1. For in vivo delivery, the genetic material is delivered directly to the patient using viral or non-viral vectors. For ex vivo delivery, the patient’s cells are extracted, then modified with the desired genetic material before being re-introduced back to the patient. A summary of some common terms in gene therapy is presented in Table 1.

**Table 1:** Common Terms and Definitions in Gene Therapy

Term	Definition
Capsid	The protein shell of a virus used as a vector for delivery of the genetic payload
Episomal	An extrachromosomal piece of DNA that can exist independently for a period of time and then integrate into chromosomes. Episomes differ from plasmids in that plasmids do not integrate into chromosomes. Episomes are not used as vectors.
Plasmid	Extra-chromosomal, self-replicating (independently of normal chromosomal DNA), double-stranded, circular DNA molecules, usually found in bacteria. Plasmids are not necessary for the survival of bacteria under normal conditions. They generally carry only a small number of genes, notably some associated with antibiotic resistance. Whereas plasmids carry the genes for self-replication, episomes do not. Plasmids may be used as vectors.
Serotype	A serologically distinguishable strain of a microorganism. More specifically, the subtype of AAV, i.e. AAV2, AAV8, etc. The serotypes differ in their tropism.
Shedding	The process by which the viral vector is released and excreted.
Transduction	The transfer of DNA using a viral vector. Uses a biological method that infects host cells.
Transfection	The transfer of DNA without using a virus as a vector. Uses chemical and nonchemical methods like plasmids.
Transgene	A gene that has been transferred naturally, or by any of several genetic engineering techniques, from one organism to another. Simply, the gene you are transferring into humans.
Tropism	The ability of a vector to bind to and enter a specific cell type like the heart or muscle.
Vector	The system used to delivery genetic material to the nucleus which can be non-viral, like liposomes, or viral, like adenovirus or AAV.

### Virus-based gene therapy

When virus-based vectors are used for the transfer of a therapeutic gene, or a transgene, into a nucleus, the process is called transduction [3]. As a vehicle, vectors for gene therapy must ideally target the right cell with adequate onset and duration of expression, and be non-toxic [4]. Although viral vectors are attractive in gene therapy because of their higher efficiency and specificity, challenges remain in choosing a vector that is capable of meeting all requirements of the ideal vector since viral vectors are generally associated with more toxicity and higher technical demands than non-viral vectors [3, 5]. A list of vectors in gene therapy and their characteristics is summarized in Table 2.

**Table 2:** Summary of Selected Approved Gene Therapies

Viral Vectors							
Product Name (region)	**Type**	**Indication**	Dose**	**Route of Administration**	**Expression**	**Adverse Events**	**Notes**
Luxturna (US)	AAV2	RPE65 mutation-associated retinal dystrophy	1.5 × 10^11^ vg	Subretinal injection	Intermediate to long (months -years)	conjunctival hyperemia, cataract, increased intraocular pressure, retinal tear	Use in adults; Use in infants under 12 months of age is not recommended
Glybera (US* & EU*)	AAV1	familial lipoprotein lipase deficiency (LPLD)	1 × 10^12^ LPL^S447X^ gc/kg	Intramuscular	Intermediate to long (months -years)	Extremity pain; headache; fatigue; hyperthermia	High levels of vector DNA were observed up to 12 months after dosing in the target tissue for Glybera, injected leg muscle, but not in non-injected muscle
Zolgensma (US)	AAV9	spinal muscular atrophy	1.1 × 10^14^ vg/kg	IV infusion	Intermediate to long (months -years)	Reported cases of liver damage and deaths due to liver failure; thrombocytopenia; blood clotting problems;	Use in patients < 2 years of age; black box warning of Acute serious liver injury and acute liver failure
Imlygic (US)	HSV	melanoma	up to a maximum of 4 mL of 10^6^ PFU/mL	Intratumoral	Transient	Infusion-related reactions; Cellulitis	Product is a live, attenuated herpes simplex virus and may cause life-threatening disseminated herpetic infection in patients who are immunocompromised
Gendicine (China)	AV5	Head and neck cancer	10^12^ virus particles to a total of eight doses	Intratumoral	Short (weeks)	Infusion-related reactions	Difficult to find information on the product

Footnotes: * removed from the market; ** amount of empty capsids is unknown. Gene therapies related to cell-therapy, like Kymriah® and Yescarta®, have be purposefully excluded. Legend: gc, genome copies; vg, vector genomes; PFU, plaque-forming units; kg, kilogram

Viral vectors can be classified into integrating or non-integrating vectors. Integrating vectors, such as retrovirus and about 10% of AAV, can integrate transgenes into the chromosomal DNA allowing permanent expression of the transgene resulting insertional mutagenesis [4, 6]. One notable example of insertional mutagenesis can be found from a trial in which young patients developed T-cell leukemia after three years of receiving the retroviral vector-mediated cell therapy for X-linked severe combined immunodeficiency (SCID). The cancer developed due to the unexpected vector insertion into the LMO-2 gene that is known to cause childhood cancers.

**Fig. 2:**
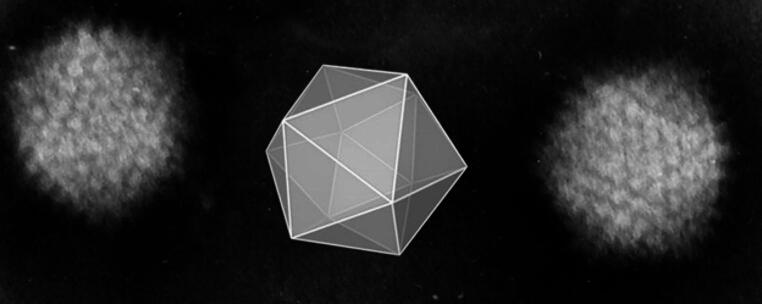
Electron microscopy of two adenoviruses and their associated icosahedral structure. Middle drawing is a cartoon commonly used to illustrate the capsule structure of adenoviruses. Figure courtesy of Graham Beards and Wikipedia Creative Commons (https://commons.wikimedia.org/wiki/File:Icosahedral_Adenoviruses.jpg)

On the other hand, non-integrating vectors such as adenovirus (AV), herpes simplex virus (HSV), and the remaining 90% of associated adenoviruses (AAV, which will be discussed later in the section on formulation; Fig. 2) are maintained as episomes, and expression of the transgene is lost during cell division resulting in a lower risk of insertional mutagenesis [6]. Immune mediated adverse events most often occur with AV vectors but can happen with any viral vectors [7]. A clinical trial conducted in 1999 at the University of Pennsylvania using the first-generation adenoviral vector expressing the OTC gene resulted in the death of a patient after experiencing systemic inflammation and multi-organ failure [8]. For this reason, assessment of the immunogenicity potential is essential during gene therapy development.

Another factor to consider when choosing a viral vector is their package capacity and tropism. Retroviral vectors can only target dividing cells while most of the other vectors, including AAV, AV, and lentivirus (a genus of retrovirus), can target both diving and non-dividing cells and can selectively target specific cells or tissues of interest by altering the vector system through pseudotyping or engineering [4]. Among different viral vectors, AAV is versatile and different serotypes can be engineered to demonstrate low immunogenicity, target specificity, and have discrete genome insertion sites [3, 9]. There are several different AAV serotypes as demonstrated in Table 3. Each AAV serotype exhibits differences in transgene expression level, tissue tropism (target tissues), and potential for immune reaction.

**Table 3:** Abbreviated Table of AAV Serotypes

Type	Transgene Expression Level	Target Tissues	% Neutralizing Antibodies	Origin
AAV1	Medium	Muscle, liver, CNS	67%	NHP
AAV2	Low	CNS, liver	72%	Human
AAV3	Low	Liver (cancer cells)	Not known	NHP
AAV4	Low	Lung	10%	NHP
AAV5	Medium	Muscle, liver, CNS, eye	40%	Human
AAV6	Medium	Muscle, liver, spinal cord	46%	Human
AAV7	High	Muscle, liver	Not known	NHP
AAV8	High	Muscle, liver, eye	38%	NHP
AAV9	High	CNS, heart, muscle, liver	47%	Human
AAV10	Medium	CNS, muscle, liver	Not known	NHP

Table modified from Nobrega, Mendonca, and Matos [17] and Boutin et al. [100]. NHP: Nonhuman primate

Disadvantages of AAV include a selection of serotypes and small package size of about 5 kb that limits the insertion of large genes [10]. However, its small size allows the in vivo injection into certain types of cells (i.e., hepatocytes and retinal cells) more favorable than other vectors. A wild-type AAV is not known to have a harmful effect on humans, but recombinant or engineered AAV vectors can induce T-cell responses, mostly due to the interaction between its capsid and cellular receptors [11]. A summary of selected approved gene therapies currently on the market can be found in Table 2.

### Plasmid-based gene therapy

Although this review will focus on the computational modeling of AAV, models developed for AAV can be translated to other types of gene therapy delivery, including plasmid-based. When transgenes are delivered by non-viral means, the process is called transfection. There are several non-viral vectors, with plasmids being one of them. According to Gene Therapy Clinical Trials Worldwide by the Journal of Gene medicine, about 15% of all clinical gene therapy trials are conducted by plasmid DNA alone without a helper viral-vector. Plasmids are circular double stranded DNA (dsDNA) (denoted pDNA) that are rapidly degraded via endocytosis when administered systemically unprotected. For this reason, they must be to be injected directly in vivo to the target site or administered systemically by encapsulating within liposomal systems such as lipoplexes or polyplexes [12, 13]. Other technical methods for delivery of transgene, such as gene gun, electroporation, nanoparticles have also been developed and used in preclinical and clinical studies [14].

Due to the inherent negative charge of nucleic-acid-based molecules (i.e., DNA and RNA), cationic lipids or polymers have been utilized to encapsulate plasmid DNA molecules to enhance the delivery into the cell membranes [12]. Although the positive charge in liposomes enhances the cellular uptake of negatively charged genetic materials, nonspecific interaction with other serum proteins or enzymes that are negatively charged can result in hemolysis or low transfection efficiency [5]. Only a portion of injected pDNA can enter the nucleus due to cellular and extracellular membranes and matrices that hinder entry. Once they reach the nucleus, pDNA often stay episomal resulting in only transient expression of the gene [12].

The major disadvantage of plasmid vectors is delivery efficiency, such as cellular uptake, but a pDNA can have high capacity of gene size and production ability, as well as low risk of immunotoxicity [12]. To overcome the challenges, a combination of pDNA with a viral vector or other technique such as electroporation, ultrasound, or microbubbles is used to improve the efficiency of delivery [15]. Initially, many trials for cardiovascular diseases had high hopes for plasmid-based gene therapy; however, more recent studies attested that due to rapid clearance with pDNA alone or accumulation of lipoplexes and polyplexes into other tissues, as well as unpredictable results with various techniques, confirmed the inefficiency even when administered directly in vivo [16].

### Interplay between formulation, route of administration, and patient factors

Although gene therapy is talked about as if it were one thing, it is not. Gene therapy is complex, and its safety and efficacy are affected by the formulation, how the formulation is administered, and by patient characteristics. It is beyond the scope of this review to present all the issues related to the interplay of these factors (the reader is referred to the Handbook of Gene and Cell Therapy for more information [17]) so what will be presented herein will be a brief overview of the topic, some of which has already been briefly discussed in Sect. 3 and will be expanded in this section.

For gene therapy to be successful, the gene or genetic material (which will be referred to hereafter as the genetic payload), whether it is encoding genes, transgene, or small-interference RNA (siRNA), must be delivered to the target, e.g., liver or muscle, it must be taken up by the cell and cross into the nucleus, and then be activated. This activation can come in many different forms:

- the genetic payload can be transcribed and then translated into a gene product replacing a genetically deficient product in a patient,
- the payload could work to silence a deleterious gene in the patient (gene silencing),
- the payload could edit a malfunctioning gene in the patient, or.
- the payload could introduce a suicide gene into the genome leading to cell death (such as for an oncolytic virus).

For lifelong effect to occur, the activation must be long-acting. Further, this entire process must occur safely and have a high rate of efficiency that translates to clinical effect, i.e., the genetic payload has the desired clinical effect.

For now, the ‘formulation’ will refer to the genetic payload, which may or may not be surrounded by a carrier, like a liposome or viral capsid, administered as a single-use suspension. The first step in the process is the administration of the formulation and delivery of the genetic payload to the target cell. Direct systemic administration of DNA or siRNA is difficult because of their low bioavailability, low ability of cross cell membranes, and limited circulation half-life due to the presence of circulating nucleases [18, 19]. Hence, a carrier is often used to augment delivery of the genetic payload; this is referred to as the ‘vector’. A vector is the system used to deliver the genetic payload to the nucleus. How that happens is one of the many different complexities of gene therapy as delivery can be either viral or non-viral in nature.

Non-viral delivery methods can be physical or chemical in nature. Physical methods use physical methods to cross cell membranes and include such methods as microneedle delivery of the material into the cell interior or microneedles that deliver material across the skin. Chemical methods are combination systems of the gene or DNA with chemical systems like liposomal delivery or polymer based nanocarriers. But by far, the major delivery vehicle for genetic payloads is viral delivery. Viruses have evolved with man and have efficient means of inserting and replicating their genetic material within the host. Viral delivery takes advantage of this by using the virus as the vehicle for delivery. There are a whole host of potential viruses to choose from: adenovirus, lentivirus, retrovirus, adeno-associated viruses (AAV), vaccinia, poxvirus, herpes virus, and others. Each of these viruses have their own safety profiles. Again, it is beyond the scope of this review to cover the pros, cons, and issues related to every virus delivery system, so this review will focus on adenovirus and AAV delivery, two of the most common viral delivery systems used today. As an aside, gene therapy has lots of jargon, like any scientific field, and two terms that are sometimes used interchangeably, but should not be, are transfection and transduction. Transduction refers to delivery of the genetic payload to the nucleus by viral mechanisms, whereas transfection refers to delivery by non-viral mechanisms.

Adenoviruses, the viruses most often associated with the common cold, are medium-sized, nonenveloped viruses with an icosahedral nucleocapsid (capsid for short) containing a double-stranded linear DNA as their genetic material (Fig. 2). Adenoviruses have been the most common payload delivery in clinical studies since their initial report in 1993. Ronald Crystal and colleagues first demonstrated the use of an adenovirus vector coding for the normal human CFTR cDNA to the nasal epithelium in a 23 year. old patient with cystic fibrosis [20]. Adenoviral vectors have many advantages including high transduction (high formation of gene product), do not integrate with host DNA, and can be produced at high levels pharmaceutically, but are limited by the transient expression of the transgene product, and they stimulate a strong immune and inflammatory response. Since adenoviruses do not integrate into their host’s DNA, they exist in the nucleus in a transient extrachromosomal state being transcribed like any other gene in the nucleus, but they are not replicated when the cell divides. Going back to the patient with cystic fibrosis, at the time of the study it was not appreciated how highly immunogenic adenovirus vectors were, and that subsequent administration of the product resulted in a waning of effect over time with repeated administration, such that by the time of the 3rd administration about 2 months later, almost no gene product could be detected. Despite many attempts to make adenovirus vectors less immunogenic, adenovirus has so far proven to be too strongly immunogenic to the human immune system. Nevertheless, adenovirus is still used today as a delivery vehicle for many gene therapies under clinical development.

AAVs were identified in 1965 and were initially thought to be a contaminant in the isolation of adenoviruses. AAVs are much smaller than adenoviruses and require a helper virus, which could be an adenovirus, for replication. AAVs were initially thought of as “satellite” viruses since their life cycle revolved around other viruses [21]. They are similar in structure to adenoviruses but have smaller capsids and carrying capacity. Many different AAV serotypes have been isolated and identified, each defined by their capsid motif proteins and the organs they target (Table 3). Because of their high seroprevalence in humans, no illnesses have been attributed to them. Although they have smaller carrying capacities, AAVs are less immunogenic, have a reduced inflammatory response, and are overall safer compared to adenoviruses.

One last factor related to formulation that may affect transduction, and possibly safety, is empty capsids [22]. Ideally, all of the gene product will be loaded into AAV capsids, but sometimes this is not the case. For any dose, some percent of the formulation will be a capsid without payload, so-called empty capsids. Empty capsids are taken up into cells but have no therapeutic benefit because they contain no payload. The role of these empty capsids on dosing, safety, and efficacy is not completely known. If capsids are responsible for the immune response to gene therapy, then a high empty capsid ratio may be leading to an unnecessarily high risk of immune response. Empty capsids may also be a safety concern, as empty capsids have been shown to increase hepatic transaminases in mice [23]. In a clinical study in 23 healthy volunteers who were administered empty capsids (n = 8) or empty capsids coadministered with tolerogenic ImmTOR nanoparticles (n = 15), no serious adverse events were noted but dosing with empty capsids did appear to induce proliferation of CD4 + and CD8 + lymphocytes which peaked about a week after dosing [24]. But there is also the argument that empty capsids may act as “effective decoys” for pre-existing antibodies to AAV capsids [25]. Whatever their role, and there will be a lot more to say about this in the future [26], it is clear that manufacturers must be able to control the process of their capsid formation so that the empty capsid ratio remains constant across lots [27].

Once you have the vector, a route of administration needs to be chosen, of which intravenous (IV), subcutaneous (SC), intra-arterial (IA), and intramuscular (IM) are the usual choices. With each route the pharmacokinetics (PK) will be different. Chen et al. [28] argued that the typical concept of PK does not “adequately describe the physiological processes that apply to [recombinant AAV] therapy” because AAV is not one thing, but many things: the AAV capsid, the genetic payload, transgene mRNA, and transgene protein. So, when discussing gene therapy PK you need to consider the PK of each of these components.

With regards to the vector, and again this will focus on viral vectors, the issue of bioavailability, biodistribution, and time course must be considered. Different routes of administration will have different bioavailability for the vector. Usually, it is stated (for small molecules) that IV administration has 100% bioavailability, but this is because it is assumed that venous concentrations equal arterial concentrations, i.e., there is no first pass from the lungs. But this is not the case with viral vectors; it is entirely possible that vector uptake and metabolism occurs in the lungs during the oxygenation of venous to arterial blood. However, this is highly dependent on the AAV serotype as demonstrated by Zincarelli C, et al. [29]. Hence, IV may not be 100% for all viral vectors. Only IA administration should have 100% bioavailability, and all others are expected to be less than that.

With each route, the biodistribution of the vector may change and with this change in biodistribution, an effect on transduction efficiency occurs as well. For example, Huard et al. [30] reported that transduction efficiency was higher in the heart, diaphragm, and intercostal muscles after intra-arterial administration of adenovirus vectors into the left cardiac ventricle compared to IV administration, but liver showed higher transduction after IV administration. Another example of the role route of administration plays in delivering gene products to key regions is with expression in the central nervous system [31]. Burstein and colleagues reported that the biodistribution of AAV9 into the CNS and peripheral nervous system (PNS) differs by the route of administration in cynomolgus monkeys. Their study compared different routes: three different intrathecal routes, intravenous, and intraocular administration. AAV9-CLN5 was administered as a single dose and biodistribution was studied 30 days later. Intracerebroventricular (ICV) and intracisternal magna (ICM) administration achieved broad and comparable distribution across most brain regions, whereas intrathecal and intravenous administration resulted in 10- to 100-fold lower levels of AAV9 in these same brain regions. All routes of administration resulted in similar levels in the PNS. That the route of administration affects the biodistribution of the vector, and subsequent transgene product formation, may come as no surprise to pharmaceutical scientists involved in small molecule or biologics development, but there is a genuine sense of surprise by this observation in the early gene therapy literature.

There is a remarkable lack of pharmacokinetic time-course data for viral vectors, but it would be expected that the time-course of the vector concentration changes with route of administration and with different serotypes, especially comparing intravenous or intra-arterial administration to intramuscular administration. Ni and colleagues [32] reported peak blood copy numbers 1 day after intramuscular administration of rAAV1 or rAAV8 in mice, whereas Kotchey and colleagues [33] reported peak copy numbers 1–2 h after intravenous administration of rAAV8 in mice.

Route of administration is also an important factor in safety through its off-target drug effects. For example, suppose the gene target is the muscle and the route of administration is intravenous or intra-arterial. Administration by these routes will invariably lead to significant distribution to non-muscle tissues. For example, the AAV9 capsid attaches to an unidentified glycan receptor in muscle for transfection to occur, but high levels of transduction have also been observed in liver and heart, which may lead to off-target safety effects. This is also borne out by epidemiological analysis – different routes of administration have different safety profiles for viral vectors. Kuzmin et al. [34] analyzed data from 149 clinical trials using AAV. Of these, 21% reported an administration-related adverse event, which was defined as an adverse event within 28 days of administration. IV administration was deemed the safest route with only 12% of studies reporting an administration serious adverse event, compared to 27% for intramuscular and 33% for intra-arterial.

In addition to formulation and route of administration, patient factors may affect transduction efficiency. Davidoff et al. [35] showed that male mice when dosed with AAV2 or AAV8 had 5- to 13-fold higher expression of the target protein in male mice compared to females, which may be a differential effect of androgens. Maguire et al. [36] also showed that females expressed greater protein expression than males after AAV9 administration in two different mice species. Surprisingly, whether these sex effects can hold in humans has not been studied. Age has also been shown to play a role – at least in animals. Polinski et al. [37] showed that protein expression was lower in aged rats compared to younger rats, which did not appear to be capsid mediated since the experiment was repeated using different capsids: AAV2, AAV5, AAV9, and lentivirus. Laz and Tuszynski [38] delivered AAV9-eGFP and AAV-PhP.eB-eGFP intrathecally or intravenously to aged and juvenile mice. Aged mice intravenously dosed with AAV9 had a reduction in brain protein expression compared to young mice. Surprisingly, they found no difference when AAV9 was administered intrathecally or using intravenous AAV.PhP.eB-eGFP.

Another patient-specific factor that could affect the safety and efficacy of a gene therapy product is the prevalence of preexisting neutralizing antibodies, which neutralize the effect of a gene therapy through the action of antibodies against the viral capsid. Adenoviruses and AAVs are modified viral capsules. It’s entirely possible for broad segments of a population to already have been exposed to a recombinant adenovirus or AAV capsid that is similar enough in nature, prior to receiving treatment, and the body has developed antibodies to them (innate immunity). Table 3 shows the prevalence of pre-existing neutralizing antibodies in the population. Once the treatment is administered, the body may also develop antibodies against the delivered capsid, which may prevent future repeated administration of the vector from working (adaptive immunity). In fact, repeated administration will prime the immune system making it better and better at attacking the capsid and neutralizing it.

The presence of these neutralizing antibodies starts early in life and is long-lasting. Calcedo et al. [39] demonstrated that neutralizing antibodies to AAV2 and AAV8 (in the absence of any gene therapy) are moderate at birth, decrease between 7 and 11 months of age, but progressively increase thereafter through adolescence. In 2006, 7 men received gene therapy using rAAV2 to treat hemophilia B. The trial never progressed because the effect of the therapy was short-lived; by 14 weeks the Factor IX activity was no longer detected [40]. Over a decade later, the same investigators brought those patients back and tested them for neutralizing antibodies to the therapy they received [41]. Three subjects demonstrated persistent neutralizing antibodies of high titer to AAV2. Because of the high degree of conservation among AAV capsids in the amino acid sequence the patients demonstrated cross-reactivity to AAV5 and AAV8. Even though patients received AAV2, over time they developed a polyclonal response to AAV5 and AAV8. This means in these patients, even if a new gene therapy for hemophilia is developed, they most likely won’t be able to benefit from it because of their neutralizing antibodies.

Are there other patient-specific factors that may affect product expression after gene therapy? Quite possibly. Could there be drug interactions with small molecules or biologics to viral vectors? Maybe. It seems unlikely, but it would be naïve to say, ‘absolutely not’. We used to believe, back when monoclonal antibodies (mABs) were first introduced, that there would be no drug interactions with small molecules because of the high specificity MAbs exhibit for their target. We now know that mABs targeting T-cells can affect cytokine expression, which in turn can affect cytochrome p450 expression, which in turn can affect a drug’s metabolism, such that a mAB can have a drug interaction with a small molecule under certain conditions. Can the reverse be true? Can a small molecule affect gene therapy?  Could prior administration of an immunosuppressant like tacrolimus suppress the immune response and decrease the effect of neutralizing antibodies, possibly improving the efficacy of gene therapy? What about a patient’s weight? Could obese patients respond differently to gene therapy? Obesity induces a low-level inflammatory state. Could this low-level inflammatory state affect expression of the target protein? Maybe. And what about race? Could a patient’s race affect gene therapy? Could there be a different efficacy and safety profile between Japanese and those of Western descent? Any of these could be possible. Gene therapy is too new at this time for all of the factors affecting its safety and efficacy to be known.

### Regulatory guidance

Although at the time of writing there are no regulatory or organizational guidances on the modeling of gene therapies, there are several industry guidance documents available to advise companies on the pre-clinical and clinical studies recommended for approval and to help guide model development. Some of the guidance documents relevant to gene therapy are summarized below in Table 4. Of note, in addition to guidance on gene therapy, there may also be guidance documents related to specific diseases; however, in the interest of brevity only general guidances as they pertain to gene therapy development have been listed. It is important to familiarize oneself with the studies conducted and required for regulatory submission as indicated by the guidances.

**Table 4:** Summary of Guidance Documents Available for Gene Therapy Development as of 2022

Title	Organization	Year	Scope
Human Gene Therapy Products Incorporating Human Genome Editing	FDA/CBER	2022	Developing products incorporating genome editing
Interpreting Sameness of Gene Therapy Products Under the Orphan Drug Regulations	FDA/CBER	2021	Factors to consider when determining sameness for gene therapy products
Studying Multiple Versions of a Cellular or Gene Therapy Product in an Early-Phase Clinical Trial	FDA/CBER	2021	For gathering safety and activity of multiple product versions during a single trial
Long Term Follow-up After Administration of Human Gene Therapy Products	FDA/CBER	2020	Trial design of long-term follow-up studies
Testing of Retroviral Vector-Based Human Gene Therapy Products for Replication Competent Retrovirus (RCR) During Product Manufacture and Patient Follow-up	FDA/CBER	2020	Testing for RCR during the manufacture and during follow-up monitoring
Recommendations for Microbial Vectors Used for Gene Therapy	FDA/CBER	2016	IND submission for microbial vectors used for gene therapy (MVGTs)
Design and Analysis of Shedding Studies for Virus or Bacteria-Based Gene Therapy and Oncolytic Products	FDA/CBER	2015	How to conduct shedding studies during preclinical and clinical development
Considerations for the Design of Early-Phase Clinical Trials of Cellular and Gene Therapy Products	FDA/CBER	2015	Designing early-phase clinical trials for the primary objectives of safety, tolerability, or feasibility
Preclinical Assessment of Investigational Cellular and Gene Therapy Products	FDA/CBER	2013	Substance and scope of preclinical information needed to support clinical trials
Clinical Considerations for Therapeutic Cancer Vaccines	FDA/CBER	2011	Considerations common to early phase and late phase clinical development of therapeutic cancer vaccines
Potency Tests for Cellular and Gene Therapy Products	FDA/CBER	2011	Recommendations for developing tests to measure potency
Quality, Preclinical and Clinical Aspects of Gene Therapy Medicinal Products	EMA	2018	Defines scientific principles and guidance for the development and evaluation of gene therapies
Development and Manufacture of Lentiviral Vectors	EMA	2005	Quality aspects relevant for lentiviral vectors
Non-Clinical Studies Required Before First Clinical Use of Gene Therapy Medicinal Products	EMA	2008	Focuses on the non-clinical studies required before use of gene therapies in human subjects
Follow-up of Patients Administered with Gene Therapy Medicinal Products	EMA	2009	Specific aspects of the active clinical follow-up of patients receiving gene therapies
WHO Considerations on Regulatory Convergence of Cell and Gene Therapy Products	WHO	2021	Overview of WHO’s considerations for regulation of cell and gene therapy products

### Bioanalytical considerations

By now, everyone knows the story of how Kary Mullis invented polymerase chain reaction (PCR) in the 1980s. One of the most interesting scientific figures in the last century, Dr Mullis initially had the idea for PCR while driving one night as he drove through the hills in Northern California – a classic “Eureka” moment in science. Initially dismissed by his colleagues at Cetus Corp. because the idea seemed perhaps too simple, he had success in December 1985 and the rest is history. His original paper has been cited more than 17,000 times [42]. Like any groundbreaking achievement there was debate as to whether he truly “discovered” PCR, and there were lawsuits trying to bust Cetus’ patent on the method, but nevertheless Dr. Mullis won the Nobel Prize for Chemistry for his discovery in 1993.

The idea behind PCR is to first heat DNA so the DNA in the sample separates into its individual strands. Taq polymerase, a heat-resistant enzyme which has been added to the system, builds two new double strands – one strand being an original strand and the other being a copy. Think of this as molecular photocopying. The process is then repeated many times creating multiple copies of the original sample creating as many as a billion copies of the original DNA sample in just a few hours. While revolutionary, PCR has its limitations. PCR is useful to determine whether a particular stand of DNA is present in a sample, like with genotyping, paternity testing, or molecular sequencing, but cannot tell you how much is present.

Quantitative PCR (qPCR), sometimes called real-time PCR, was developed by Russell Higuchi and colleagues at Roche Molecular Systems and Chiron to quantify the amount of a particular DNA sequence present in a sample [43]. By adding a fluorescent dye to the sample that stains nucleic acids and fluoresces upon binding, a fluorometer can be used to quantify the fluorescence of a sample and when compared to standards of known quantity, an estimate of the amount of DNA in a sample can be obtained. Hence, one of the major uses of qPCR is quantitative measurement of gene expression. Today, qPCR is a mature technique and entire books can be written on its use. Simply stated, there are a variety of different instrumentation manufacturers that put their own flavor on the basic method, e.g., TaqMan by Applied Biosystems and LightCycler by Roche, and can process thousands of samples for different probes simultaneously (multiplexing) in a day.

A new competing method has recently appeared – droplet digital PCR (ddPCR). Both qPCR and ddPCR are useful to measure gene expression. qPCR has a wider dynamic range and process more samples than ddPCR, but ddPCR is much more precise and provides absolute quantitation (does not need calibration curves). Like PCR, ddPCR uses a Taq polymerase to amplify DNA fragments but ddPCR partitions the reaction into thousands of droplets and the fluorescence of each droplet can then be counted (each drop is either negative or positive). ddPCR and qPCR give identical results when there are low levels of contamination [44].

When using AAV as the viral vector, the results from a qPCR or ddPCR are usually reported as a “physical titer” in viral genomes per mL (vg/mL) or vector copy number per mL (VCN/mL, which is equal to the vg standardized to the mass of the genomic DNA input or some housekeeping gene, i.e., vg/dg, where dg is the diploid genome). VCN is often used interchangeably with genome copies (gc/mL or GC/mL). Other types of gene therapy might report different numerators. For example, adenovirus titers are reported as plaque-forming units per ml (pfu/mL), whereas lentivirus titers are reported as “infectious” or transducing units per mL (TU/mL). Typical values reported for vg/mL are often greater than 10^10^ and may cover a dynamic range of over 100,000. For example, Greig et al. [45] reported on the pharmacokinetics of an AAV8.TBG.eGFP vector into the saphenous vein of male cynomolgus monkeys following a dose of 7.5 × 10^12^ genome copies (GC) per kg at a concentration of 7.5 × 10^12^ GC per mL over intervals that varied from 1, 10, and 90 min. Peak concentrations were on the order of 10^12^ vg/mL and by 100 h had decreased to about 100 vg/mL, a drop of ten billion! Readers looking for additional guidance or resources on bioanalytical considerations for AAV and other gene therapies can refer to the publications by Gorovits B, et al. and Kavita U, et al. [46, 47].

### Modeling platforms and techniques

Model informed drug development (MIDD) is a process regularly employed throughout the development lifecycle of a drug to expedite development, enhance scientific understanding of the drug, and ultimately ensure drug benefit is maximized for patients [48]. Computational modeling approaches in gene and cell therapy have become possible with the increased understanding of cellular and genetic processes and advances in processing capabilities of computers. Classical modeling approaches for small molecules and PK are not always applicable to gene therapies. Therefore, several modeling approaches have emerged to capture and explain these types of complex therapies.

One such methodology is quantitative and systems pharmacology (QSP). QSP or more generally, systems pharmacology, is a discipline that uses mathematical and computational modeling with a goal “to understand, in a precise, predictive manner, how drugs modulate cellular networks in space and time and how they impact human pathophysiology” [49]. QSP models endeavor to represent the biological system, including cellular and receptor interactions if needed, to answer the question of interest, leading to a better representation of physiological changes following cell and gene therapies. QSP has been applied in several cases to support MIDD [50]. Models focusing on viral diffusion or immune biology response were previously reported [51–54]. In these early reports, gene therapy related to immune cancer therapy through treatment with oncolytic viruses or combination therapy with dendritic cells were explored computationally.

Another type of modeling approach utilized for cell and gene therapy is agent-based modeling (ABM), which is a system-based modeling approach where a collection of autonomous decision-making entities called agents make decisions on the basis of a set of rules [55]. The agents collectively represent the desired biological system and provide confirmative and/or predictive results about the system. Predicting cancer and tumor growth is one area that has been explored extensively using ABM [56]. Modeling in infectious disease and viral behavior within tissues has also been explored using ABM [57].

Mechanistic pharmacokinetic – pharmacodynamics (PKPD) modeling is applicable when a biomarker or pDNA is observable systemically. A well-characterized biomarker assay can be incorporated into mechanistic PKPD models [58]. Plasma biomarkers have been explored for use in modeling most extensively in oncology [59], but exploration has begun in other areas as well such as viral vectors/plasmids. Mechanistic modeling was explored for HSV distribution in solid tumors [60]. In this report, diffusion reaction equations were used to identify factors to improve vector efficiency.

The remainder of this section will focus on the combination of PK modeling, particularly physiologically based pharmacokinetic (PBPK) modeling, and systems pharmacology for the application of modeling AAV therapies. PBPK is an MIDD approach that incorporates physiological and biochemical principles, like organ size, organ blood flow, how the organs are connected, degree of drug ionization, protein binding, and lipophilicity, to name a few, to explain a drug’s pharmacokinetics. Applications of PBPK include first-in-man dose selection, prediction of drug interactions, extrapolation of adult doses to children, prediction of drug exposure in patients with renal or hepatic impairment, and fetal exposure in pregnant women [61]. No public applications for PBPK being applied in gene therapy clinical development exist to the authors’ knowledge. However, PBPK models have been published that could help guide development of gene therapy PBPK models due to overlapping biological mechanisms; some examples include a PBPK model exploring antibody ocular disposition could guide model development for AAV therapies administered ocularly [62], a cell therapy PBPK model with tissue level parameters that can be repurposed for AAV PBPK models [63], and a PBPK model of siRNA therapy that showcases how mRNA measurements can be incorporated into model calibration [64].

Additionally, a recent meta-analysis across AAV usages in clinical settings found there still exists a lack of dose-toxicity and dose-efficacy data that causes AAV dosing regimens to remain elusive [65]. As such, there exists a large unmet opportunity to standardize PBPK strategies to support gene therapy dose selection and clinical trial design. As such, this section focuses on recommendations for applying PBPK modeling to support AAV-based gene therapy development.

**Fig. 3:**
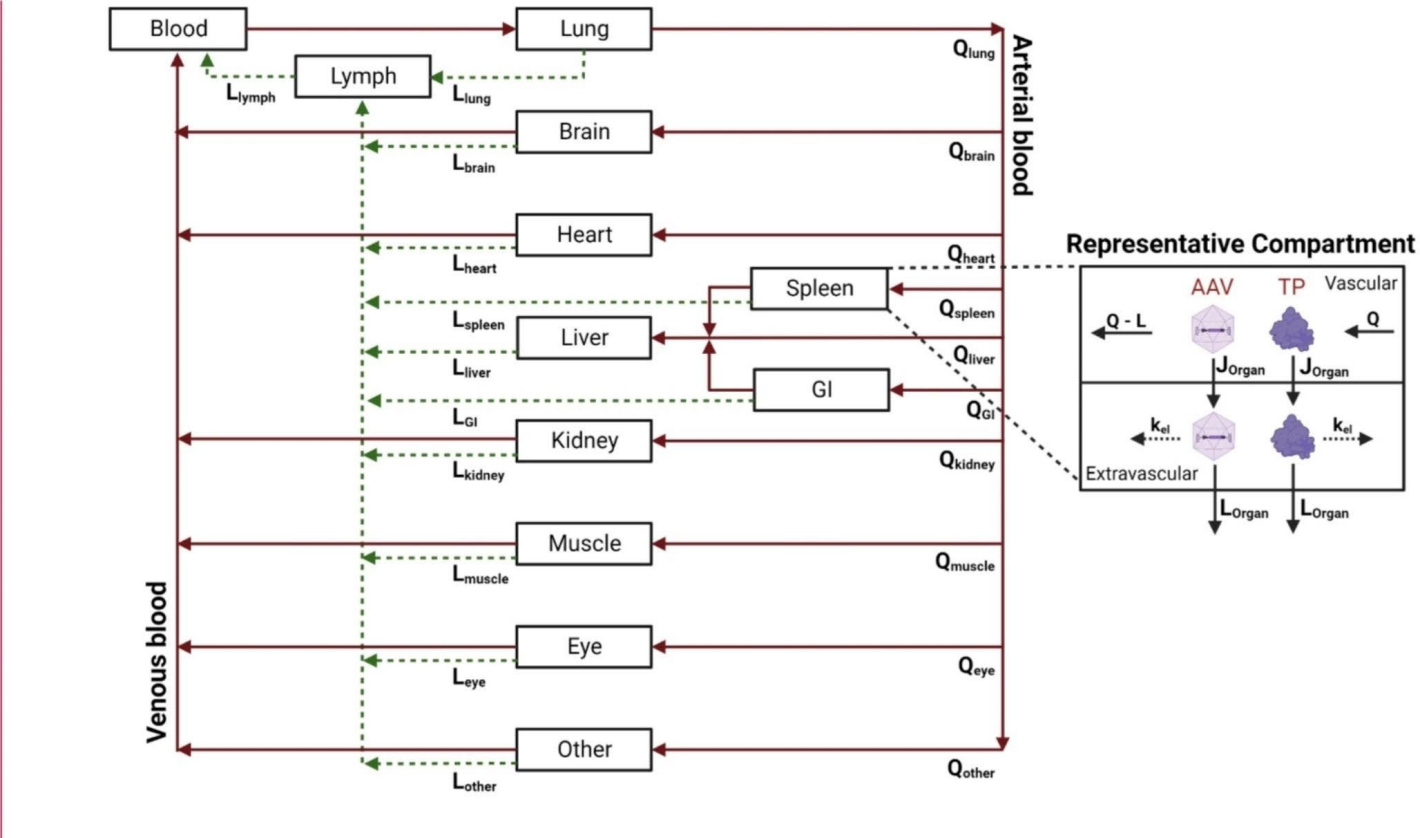
Example schematic of systemic AAV PBPK distribution with suggested applicable compartments based on AAV tropism and frequently targeted tissues of AAV therapy. Applicable modeling parameters as referred to in Table 5. Created with BioRender.com

PBPK models seek to mathematically represent absorption, distribution, metabolism, and excretion (ADME) of administered therapies, often to support dose optimization [66]. Due to the mechanism of action through which AAV-based gene therapies work, we recommend PBPK modeling for AAV-based gene therapies include ADME considerations for both (1) the AAV capsid and (2) the expressed protein. In addition, it may be necessary to consider the effect of the empty capsid, as mentioned earlier. As such, we recommend PBPK modeling for AAV-based gene therapies include multiple biological processes and compartments as depicted in Fig. 3, which would require the model parameters listed in Table 5. Briefly, these biological processes include AAV biodistribution, AAV cellular uptake, uncoating and transduction of packaged gene, and biodistribution, relevant pharmacodynamic effects, and elimination of the transduced protein. Here considerations for modeling these biological processes will be explored in greater detail.

**Table 5:** Key biological processes driving AAV-based gene therapy PK and PD. Potential in vitro or in vivo assays that can be used to characterize model parameters describing these key processes are given

Biological process	Model parameter	Assay	Examples
PBPK Distribution			
Tissue-specific blood flow	Q_tissue_		
Tissue-specific lymph flow	L_tissue_		
Tissue-specific first order transmigration rate	J_organ_ (AAV, TP)	Transendothelial migration assay	[101]
In vitro			
AAV-AAVR binding and internalization			
AAV - AAVR binding affinity	k_on_/k_off_	SPR, ELISA	[102]
AAVR surface expression	R_max_	Flow cytometry, Imaging	[102]
AAVR synthesis rate	k_syn, AAVR_	Flow cytometry, Imaging	None identified
AAVR internalization rate	k_int,AAV−AAVR_	Flow cytometry, Imaging	[103, 104]
AAV endosomal escape	k_escape_	Western blot	[105]
AAV intracellular localization	k_endo_, k_nuc_	Western blot	[106]
DNA dynamics			
Nuclear AAV uncoating/DNA release	k_uncoat_	Imaging, ELISA	[107]
Degradation of released DNA	k_deg, DNA_	Stability assays	[93]
DNA transcription rate	k_tc_	qPCR	[108]
mRNA export from the nucleus	k_nuc mRNA_	Western blot	[109]
mRNA translation rate	k_tl_	qPCR, Western blot, ELISA, LC/MS	[110]
In vivo			
AAV PK			
AAV systemic concentration	AAV PK parameters	LC/MS, qPCR	[33]
AAV tissue concentrations	σ, AAV	IHC, LC/MS	[25, 111]
Expressed target protein PK			
Expressed protein systemic concentration	TP PK parameters	LC/MS	[25, 111]
Expressed protein tissue concentrations	σ, TP	IHC, LC/MS	[25, 111]
Efficacy			
Biomarker responses	Variable by therapy	Variable by therapy	[112]
Clinical endpoints	Variable by therapy	Variable by therapy	[112]

SPR: surface plasmon resonance. ELISA: enzyme-linked immunoassay. qPCR: quantitative polymerase chain reaction. LC/MS: liquid chromatography-mass spectrometry. IHC: immunohistochemistry

### PBPK modeling of pDNA

**Fig. 4:**
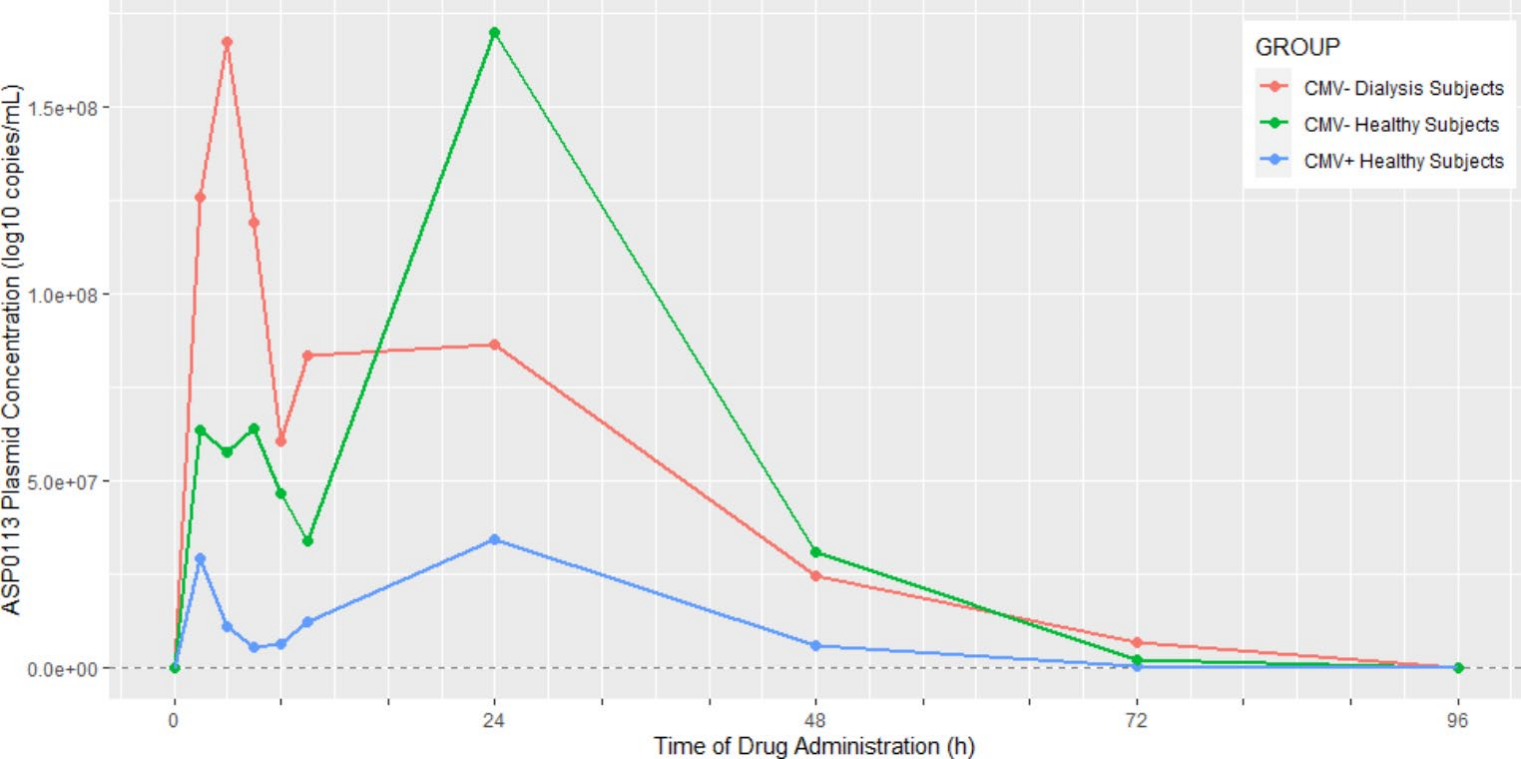
ASP0113 plasmid concentrations. Mean (SD) ASP0113 plasmid concentration in CMV-seropositive (CMV+) and CMV-seronegative (CMV–) healthy subjects and CMV-seronegative dialysis patients measured by polymerase chain reaction. Time values have been jittered for each analysis point for graphical presentation only to distinguish among the subject groups. Values below the limit of quantification were treated as 0 for calculation purposes. CMV indicates cytomegalovirus; LLOQ, lower limit of quantification. Data reported in Bonate et al. [68]

There have been few studies where pDNA concentrations were quantified over time, and even fewer in humans [67]. One of the few human reports of pDNA pharmacokinetics was by Bonate et al. [68] who reported on ASP0113, a pDNA vaccine against cytomegalovirus, in dialysis patients and healthy volunteers after intramuscular dosing (Fig. 4). What was interesting about that study was the observation of double peaks after pDNA dosing, with the second peak having higher concentrations than the first peak. The authors rationalized that the first peak was due to rapid absorption from the injection site and the later peak was due to lymphatic absorption since it occurred much later. There have been no reports of PBPK models for pDNA gene therapy products.

### PBPK modeling of AAV capsids

**Fig. 5:**
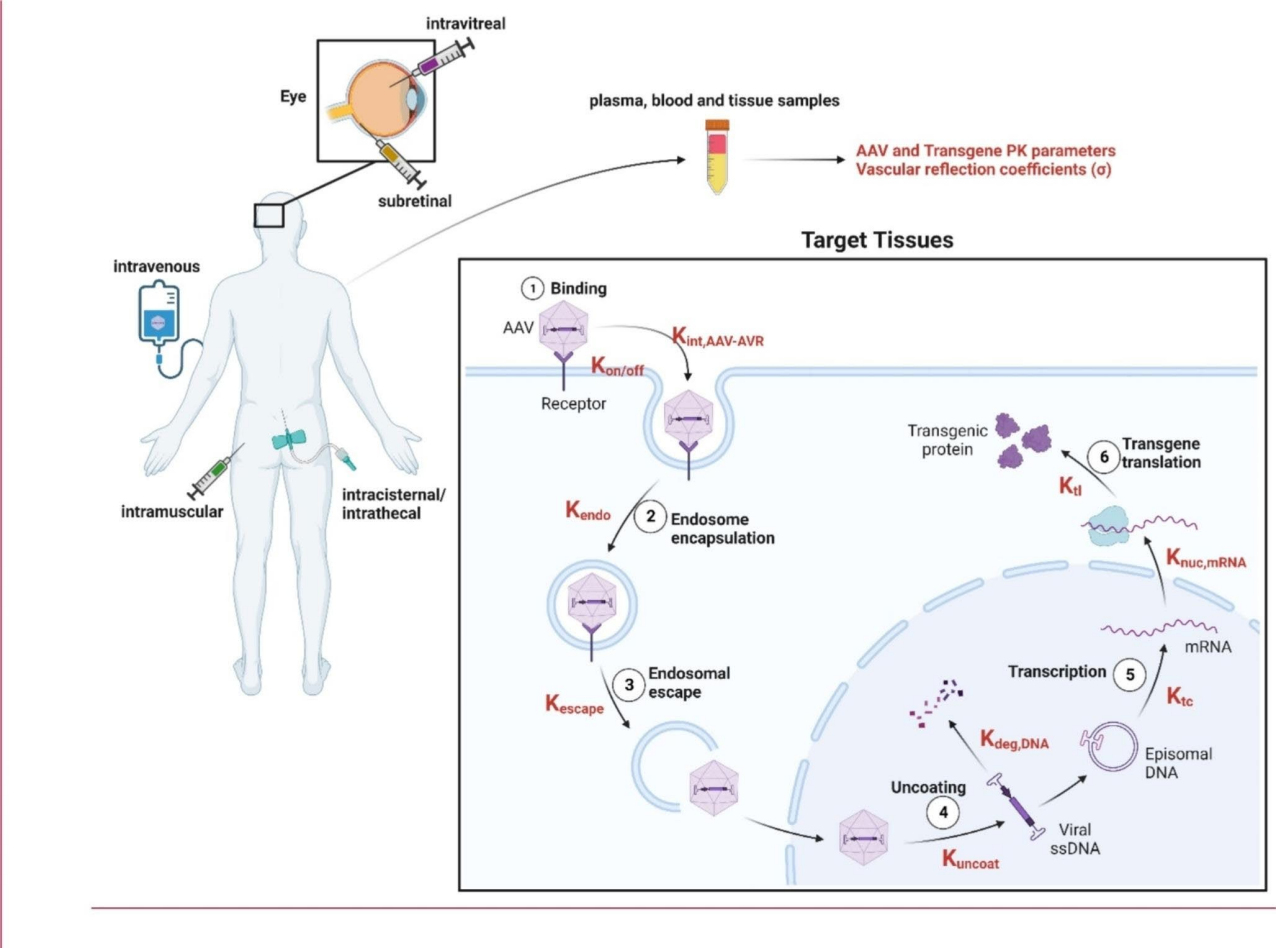
Graphical schematic of AAV cellular behavior and physiology with various routes of administration. Example schematic of systemic AAV PBPK distribution with suggested applicable compartments based on AAV tropism and frequently target tissues of AAV therapy. Applicable modeling parameters as referred to in Table 5. Created with BioRender.com

Despite the lack of published PBPK models for AAV gene therapy, it is possible to envision what such a model must look like and entail which is show in Fig. 3. The compartments included represent the commonly targeted tissues for AAV gene therapies and are driven by the tropism of the available AAV serotypes. Therefore, Fig. 3 graphically represent an example schematic of a platform-based model design that allows translation to many different types of AAV therapies. To expand on the PBPK distribution, the mechanistic behavior of the AAV once it reaches the targeted cells is depicted in Fig. 5. Therefore, to develop an AAV PBPK-mechanistic model, we recommend the model scope include the following for AAV capsids: biodistribution of the administered AAV capsids across tissues (Fig. 3), intracellular uptake through receptor-mediated endocytosis or pinocytosis, escape from endosomes into cytoplasm, uptake from cytoplasm into the nucleus, and nuclear uncoating (Fig. 5). The distribution and compartments illustrated in Fig. 3 can be adapted to different types of AAV by removing or adding compartments as they apply to the specific case. Since different AAV serotypes exhibit different tropism, accounting for serotype specific receptor parameters is necessary [69, 70]. Such parameters include AAV-AAV receptor (AAVR) binding affinity, AAVR expression across tissues/cell types, and AAVR synthesis and internalization rates (Table 5) would affect capsid uptake across tissues. A recent review by Chowdhury et al. describes how such AAVR parameters are critical towards dictating AAV tropism and their importance in PK/PD considerations [71]. Considerations specific to how the capsid is engineered may need to be modeled; for example, engineered AAV variants can also affect AAVR binding and tropism [72, 73]. It is important to note that AAVR-independent pathways, such as micropinocytosis, may be used to facilitate AAV cellular uptake; however, there are diametric findings on the role micropinocytosis plays on AAV transduction and may be serotype or cell type specific [74, 75]. Thus, we propose PBPK modeling focuses on AAVR-dependent endocytic uptake (Fig. 5; Table 5).

Once AAV capsids are endocytosed, they undergo translocation from early endosomes ultimately to the nucleus where they are uncoated. While the mechanism of nuclear entry remains not fully defined, evidence indicates that AAV capsids residing in early endosomes are sorted towards the trans-Golgi network along a retrograde transport pathway. Within the trans-Golgi network capsid conformational changes occur that allow export to the cytoplasm followed by nuclear import [76–78]. Interestingly, endocytosed AAVs do not seem to undergo recycling to the cell membrane [79] and typically escape from early/late endosomes prior to trafficking to lysosomes [80–82]. Therefore, models for intracellular trafficking of AAV capsids are proposed to include AAV escape from endosomes into the cytoplasm followed up uptake into the nucleus. Modeling AAV escape from endosomes as a single rate is beneficial as it reduces model complexity and allows the trans-Golgi network processes to be represented by a single model parameter (Table 5), reducing likelihood of model identifiability issues.

The eye compartment included within the model is a special case. The retina is protected by barriers (blood-retinal barrier (BRB)) which are made of tight junctions preventing systemic absorption of proteins and other large molecules, including AAV [83]. Generally, AAV gene therapy targeted for ocular conditions must be administered intraocularly and not systemically [84–86]. However, effects from systemic administration are possible. An example of ocular effects following systemic administration of an AAV therapy was explored by Simpson CP, et al. [87]. In this study, they found that systemic delivery led to robustly transduced cells throughout the retina with limited ability to further penetrate the retina and reach the photoreceptors. For this reason, ocular exposure of systemic administered AAV should be considered where applicable. In addition, as mentioned in Sect. 7, with the help of other modeling approaches it is possible to reproduce the distribution and intracellular processes of intraocular administer gene therapies [62]. It is also important to consider the systemic exposure and systemic effects of intraocular administered AAV therapies [88, 89].

Immune response following AAV administration may be necessary to include in the PBPK model. Wild-type AAVs are common and there is a high native prevalence of neutralizing anti-AAV antibodies in the general population [90] and is serotype dependent [91] (Table 3). Anti-AAV neutralizing antibodies can interfere with therapeutic efficacy, resulting in difficulty with dose selection and subsequently increased toxicity risk [92]. One strategy to overcome decreased efficacy by anti-AAV antibodies that is gaining popularity is to purposefully co-dose capsids containing the gene with empty capsids. Evidence has shown that protein transduction is dependent on the full/empty capsid ratio, and may increase protein transduction via empty capsids acting as a decoy to anti-AAV antibodies [25], although this is currently highly debatable. Incorporating anti-AAV antibodies into the PBPK model may be necessary depending on the target population, serotype, and empty to full capsid ratio and should be carefully considered.

#### Modeling the transduced protein

Once AAV capsids are localized to cellular nuclei, capsids are uncoated, releasing packaged genes and allowing target protein transduction. AAV uncoating remains poorly understood and appears to be a rate-limiting step in a serotype and tissue-specific manner [82]. Ultimately, AAV uncoating results in release of the packaged single-stranded DNA (ssDNA), ssDNA is converted through double-stranded form to either episomal DNA or integrated into the host genome, transcribed to mRNA, and mRNA is exported from the nucleus and translated to protein (Fig. 5; Table 5). Preclinical experiments suggest that AAV derived ssDNA and dsDNA are unstable, and degradation of these is a rate-limiting step in AAV transduction efficacy [93, 94]. Conversely, once converted episomal DNA is relatively stable and can persist in tissues for years [95]. Likewise, transcribed mRNA degradation does not appear to be a significant rate-limiting step [96]. Therefore, computational models for protein transduction should include degradation on ssDNA/dsNDA, and ssDNA and dsDNA can be lumped together for model reduction and identifiability optimization.

Considerations for modeling the transduced protein will be highly dependent on the type of transduced protein and mechanism of action it exerts. Generally, once the protein is transduced it may undergo efflux from production cells into surrounding tissue and ultimately back to systemic circulation, where biodistribution, elimination, and pharmacodynamic effects continue. Depending on the expressed protein and desired outcomes to capture with the PBPK model, biological processes such as protein-transporter interactions, enzymatic metabolism, and pharmacodynamic effects can be incorporated [97]. Local concentrations of the transduced protein in specific tissues are critical to the dynamics of transporter, metabolic, or pharmacodynamic responses, and as such transduction in specific tissues based on AAV biodistribution is necessary to model. Biomarker or clinical endpoint outcomes can be incorporated into the model based on transduced protein pharmacodynamic effects as desired.

The development of an AAV PBPK model capturing the relevant biological processes would allow for multiple MIDD applications. Such a PBPK model would capture relevant biology and mechanisms of action for both the administered AAV and transduced protein. This PBPK model would be a powerful tool to support dose optimization, allowing considerations such as transduced protein concentrations or pharmacodynamic response necessary to achieve clinical efficacy. Doses achieving target protein concentration could be explored with respect to full/empty AAV capsid ratio, route of administration, or serotype tropism. Virtual populations using this PBPK model could be developed to inform clinical population selection based on projected safety/efficacy profiles; for example, PBPK modeling could inform a threshold of preexisting anti-AAV antibodies above which clinical efficacy is anticipated to be unachievable. With the recent difficulties in selecting safe and effective doses for AAV-based gene therapies seen clinically, PBPK models allowing for in-depth exploration of AAV pharmacology could be the key the field needs to treat these unmet disease populations.

### Future/gaps/limitations

There are so many gaps in the development and modeling of gene therapy products it’s difficult to know where to begin. Three areas we have identified that need immediate attention are with regards to clinical pharmacology, basic science, and safety of AAVs.

First, the concepts of clinical pharmacology in gene therapy, treating the gene therapy product as you would any other biologic, is practically non-existent. One might think that since a patient receives only a single dose of the drug that tremendous effort would be spent in getting the dose right, but in discussions with many key opinion leaders in the area, this is not the case. There is a great deal of latitude in the dose administered. Indeed, an internal review of all AAV gene therapy studies in clincaltrials.gov showed a dose range of 2 × 10^10^ to 3 × 10^14^ vg/kg (internal data not shown), a ~ 10,000 fold range. Translation of doses in animals to humans is a black art, often with the clinically used human dose being the same as a dose in animals that showed efficacy, i.e., the dose used in animals that demonstrated an effect (vg/kg) is the same dose used in humans. Much more work could be done regarding the proper dose to take into humans.

Second, with regards to building a systems pharmacology model, conceptually it is understood how gene therapy works, but many of the basic biology and parameter values needed to build such a model are unknown. For instance, the receptor that binds AAV7 to cells is unknown. Since the receptor is unknown, its density on cells is unknown, which makes models of cell uptake difficult to build. For this reason, many parameters and processes can/are lumped together during the modeling process. This leads to less parameters requiring optimization/ parameterization; however, this can lead to a model being unidentifiable. Not only is parameter identifiability an issue, but the ability for in vitro derived parameters to be translated to the in vivo setting is limited. For one example, in vivo affinities between therapeutic antibodies and their targets measure via surface plasmon resonance have been shown to not always be translatable to in vivo affinity due to the additional environmental factors and co-receptors not accounted for with in vitro settings [98]. Predicting AAV tissue distribution and systemic PK using only in vitro data is also a large challenge, as such in vitro-in vivo extrapolation requires an understanding of pharmacology [99] that is currently lacking for AAV therapies. Much more basic science is needed regarding AAV biodistribution and uptake into cells to allow AAV PBPK/QSP models to be built with high confidence.

Lastly, there have been concerns about the safety of vector-based delivery with some recent trials being put on clinical hold due to deaths and safety concerns. It has been speculated that the sheer volume of genetic material delivered could be the cause, but this is not known with any degree of certainty. Hence, more research needs to be done on the safety of vector-based products, the reasons for safety issues, and how to prevent them in the future. PBPK and QSP based models may help in this regard by better understanding how a vector-based product distributes in the body and how it is transduced to a transgene product.

## Conclusions/summary

You might have noticed that, despite what the title of this paper suggests, there isn’t a lot of detail regarding gene therapy models in this review. That’s because there are few published gene therapy models at the time of this publication (2023). There have been a few more modeling-related posters presented at meetings, but most lack details and cannot be formally evaluated. Computational models of gene therapy are an open area for research, but with a dire lack of biological data upon which to build these models, their development is problematic and will require ingenuity and model assumptions that may not be verifiable. We hope by presenting this review we will stimulate further research and publication in the area.
